# Vitamin D Receptor Polymorphism and Breast Cancer Risk: A Meta-Analysis

**DOI:** 10.1097/MD.0000000000003535

**Published:** 2016-05-06

**Authors:** Demin Lu, Lei Jing, Suzhan Zhang

**Affiliations:** From the Cancer Institute (Key Laboratory of Cancer Prevention and Intervention, China National Ministry of Education), Second Affiliated Hospital, School of Medicine, Zhejiang University, Hangzhou (DL, SZ) and Ningbo First Hospital, Ningbo, Zhejiang, China (LJ).

## Abstract

The objective was to perform a meta-analysis to summarize the available evidence from prospective nested case-control studies on the association of vitamin D receptor (VDR) polymorphism and the risk of breast cancer.

We searched PubMed, ISI web of science, EMBASE, and reference lists for included articles. Study specific odds ratios (ORs) and 95% confidence intervals (CIs) were pooled by using fixed-effect or random-effects models.

Eight studies were included in the meta-analysis. There were no association between Fok1 gene allele contrast f versus F (OR: 0.859; 95%CI: 0.685–1.079), ff versus FF (OR: 0.893; 95%CI: 0.763–1.045), recessive models ff versus FF+Ff (OR: 0.932; 95%CI: 0.796–1.092), and dominant models ff+Ff versus FF (OR: 0.899; 95%CI: 0.780–1.037). The estimated VDR polymorphism showed no significant association between Bsm1, Taq1, Apa1 polymorphism, and breast cancer risk. In the Caucasian ethnic subgroup, no association was found between allele contrast, recessive models, and dominant models on Fok1, Bsm1 polymorphism, and breast cancer risk.

VDR polymorphism (Fok1, Bsm1, Taq1, and Apa1) were not associated with the risk of breast cancer in the general population as well as Caucasian population.

## INTRODUCTION

Breast cancer is one of the most commonly diagnosed invasive malignancies and the second most common fatal cancer for women worldwide.^1^ Risk factors for breast cancer include first-degree relatives with breast cancer, extremely dense breasts, prior benign breast biopsy results, present oral contraceptive use, nulliparity, and age at birth of first child >30 years.^2^

Higher vitamin D exposure is hypothesized to prevent several cancers, possibly through genomic effects modulated by the vitamin D receptor (VDR).^3^ Laboratory investigations have suggested that the expression of the VDR might be associated with an increased risk of breast cancer.^4^ The human VDR gene, located on chromosome 12q13, includes more than 470 single-nucleotide polymorphisms (SNPs), mostly studied SNPs as following: Fok1 (rs2228570), Bsm1 (rs1544410), Taq1 (rs731236), Apa1 (rs7975232), and Poly A (rs17878969).^5^

Several recent studies (case-control studies and nested case-control studies) investigated the association between VDR polymorphism and breast cancer risk. The results were controversial. For example, Sinotte et al, Gapska et al , and McKay et al reported increased risk among ff carriers on Fok1.^6–8^ Whereas, Anderson et al reported decreased risk among ff carriers and Curran et al, Guy et al, Abbas et al, Engel et al, Rollison et al; Fuhrman et al, Mishra et al, and Shahbazi et al reported no association between ff carriers and breast cancer risk.^8–17^

For consideration case-control studies are prone to selection bias. To overcome the shortcomings of the retrospective studies, we perform meta-analysis on prospective studies.

## METHODS

### Literature Search

We systematically searched 3 databases: PubMed, ISI web of science and EMBASE for studies published in any languages (up to August 15, 2015). The searched terms used are as follows: VDR; vitamin D3 receptor; vitamin D3 receptors; 1,25-dihydroxyvitamin D3 receptor; 1,25-dihydroxyvitamin D3 receptors; calcitriol receptor; calcitriol receptors; cholecalciferol receptor; cholecalciferol receptors; 1,25-dihydroxycholecalciferol receptor; 1,25-dihydroxycholecalciferol receptor; VDR combined with breast cancer; breast carcinoma; breast neoplasm; breast neoplasms; breast tumor; breast tumors; mammary cancer; mammary carcinoma; mammary carcinomas; mammary neoplasm; mammary neoplasms; mammary tumor; mammary tumors; FokI; BsmI; ApaI; TaqI; Cdx2, and polyA. The search was restricted to studies of human participants. We also have reviewed the reference lists of enrolled articles to identify additional articles. Ethical approval was not necessary. Because this was a meta-analysis it involved no direct handing of personal data or recruitment of subjects.

### Inclusion Criteria

For inclusion, the studies had to have met the following criteria: (1) breast cancer cases were medically confirmed pathologically; (2) the study was designed as prospective nested case-control or cohort study; (3) providing the data of VDR gene polymorphism and incidence of breast cancer; (4) detailed data of odds ratios (OR) with 95%CI; (5) all of the cases were adult; and (6) all SNPs were in Hardy–Weinberg equilibrium (HWE) (*P* >0.05). Reviews, retrospective case control studies or studies with insufficient data were excluded. When there were multiple published reports from the same study population, the most recent or the most informative report was selected for analysis.

### Data Extraction

We extracted the following information from each study: authors’ name, year of publication, study name, ethnicity, source of control, genotyping method, sample size, studied polymorphism, adjusted OR and 95% confidence interval (95% CI), and adjustments for potential confounding. When the studied population was >95% Caucasian, we included this study in the Caucasian group.

### Credibility of Meta-Analysis Results

We used Venice interim criteria to access the cumulative evidence of the genetic association between the VDR polymorphism (Fok1, Bsm1, Taq1, and Apa1) and the risk of breast cancer. The Venice interim criteria included in amount of evidence, replication of results, and protection from bias.^18^ With regard to assessment on amount of evidence, category A required a sample size >1000, category B corresponded to a sample size of 100 to 1000, and C corresponded to a sample size <100. Sample size referred to the total number of cases and controls when the least frequent genotype was used. To assess replication, when *I*^*2*^ <25% category A was given, B for 25% ≤ *I*^*2*^ ≤50% and C for *I*^*2*^ >50%. To assess protection from bias, all of the following criteria were required: (1) clear phenotype definition; (2) reliable genotyping test method; and (3) no change of result when the small sample study was excluded.

### Statistical Analysis

For each study, the HWE of SNPs in the control group by using the chi-square test was reported by original article. The multivariate adjusted ORs and 95% CIs presented in the literature were used.

Statistical heterogeneity among studies was tested with the Cochrane Q statistic, and statistical inconsistency was quantified with the *I*^*2*^ statistic.^19^ When *I*^*2*^ was from 0% to 40% along with *P* >0.10, the heterogeneity might not be important. If the meta-analysis has no heterogeneity, fixed-effects model with the Mantel–Haeszel method^20^ would be used to combine the individual studies, otherwise, the random-effects method^21^ was used for pooling.

The Egger's regression test^22^ and Begg–Mazumdar test^23^ were used to assess for publication bias. *P* <0.05 was considered statistically significant publication bias.

All reported *P*-values were two sided. All statistical analyses were performed using STATA (version 11.0; StataCorp, College Station, TX).

## RESULTS

### Literature Search

**FIGURE 1:**
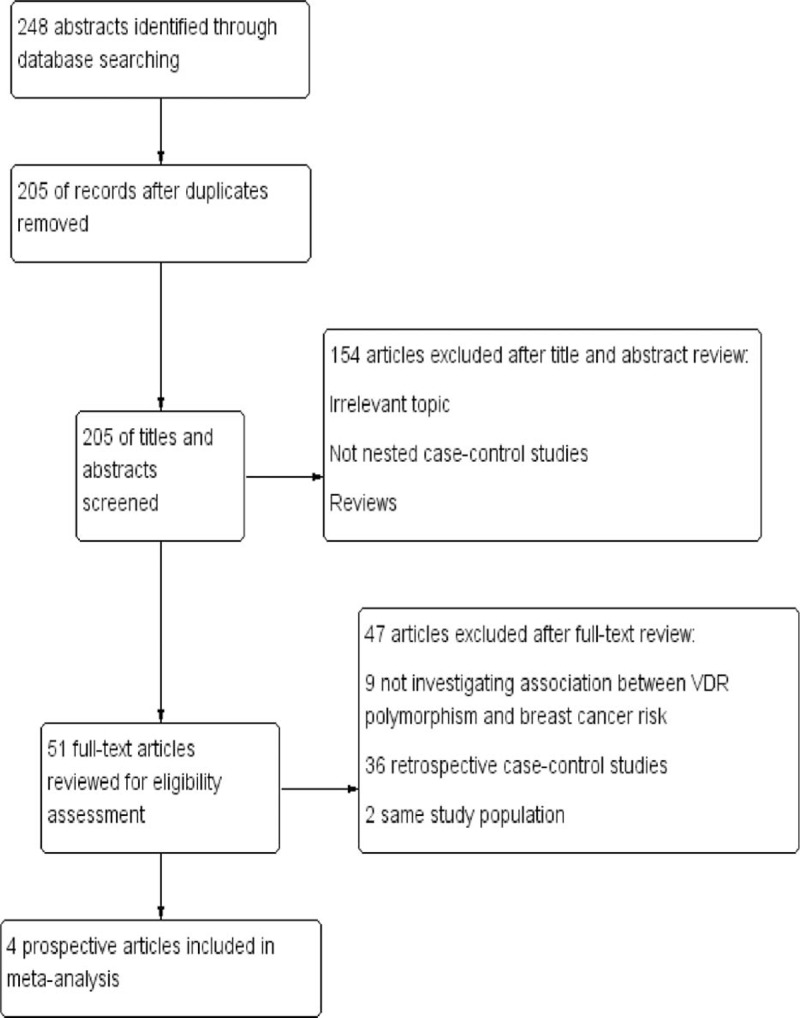
Flowchart of selection of studies for inclusion in the meta-analysis.

A total of 248 abstracts were retrieved though PubMed, ISI Web of Science, and EMBASE. After removing duplication, 205 abstracts remained. Of these 205 abstracts, we identified 51 potentially relevant studies that described the association between the VDR gene polymorphism and breast cancer risk after screening the titles and abstracts. For further assessment, 47 articles were excluded after full-test review. Nine articles were excluded because they did not investigate the association between VDR gene polymorphism and breast cancer risk, 2 were duplicate reports on the same study population, and 36 were not nested case-control studies. The study of Mckay et al contained 5 different populations, thus, it was extracted into 5 individual studies.^8^ Eight independent nested cast-control studies were eligible for our meta-analysis.^8,13,15,24^ The flow diagram of our systematic literature search is shown in Figure 1.

### Study Characteristics

The characteristics of the eligible studies in this meta-analysis are summarized in Table 1. These 8 studies were all prospective nested case-control studies published from 2000 to 2013. One article reported the data from 5 different populations. So, this article was looked at as five individual studies. We did not include the ORs of Fok1 and Bsm1 in the McCullough et al's^24^ study because the study population was the same as the Mckay et al's^8^ study. This meta-analysis included 8 prospective studies. Among these articles, all studies were conducted in the United States. Seven studies on Fok1, 6 son Bsm1, 2 on Taq1, and 2 on Apa1 were included in the meta-analysis. Ethnic subgroups were also reported on: 5 studies reported on Caucasians, 1 study on Hispanics, 1 on African Americans, 1 on Asians, and 1 on Hawaiians.

**TABLE 1:**
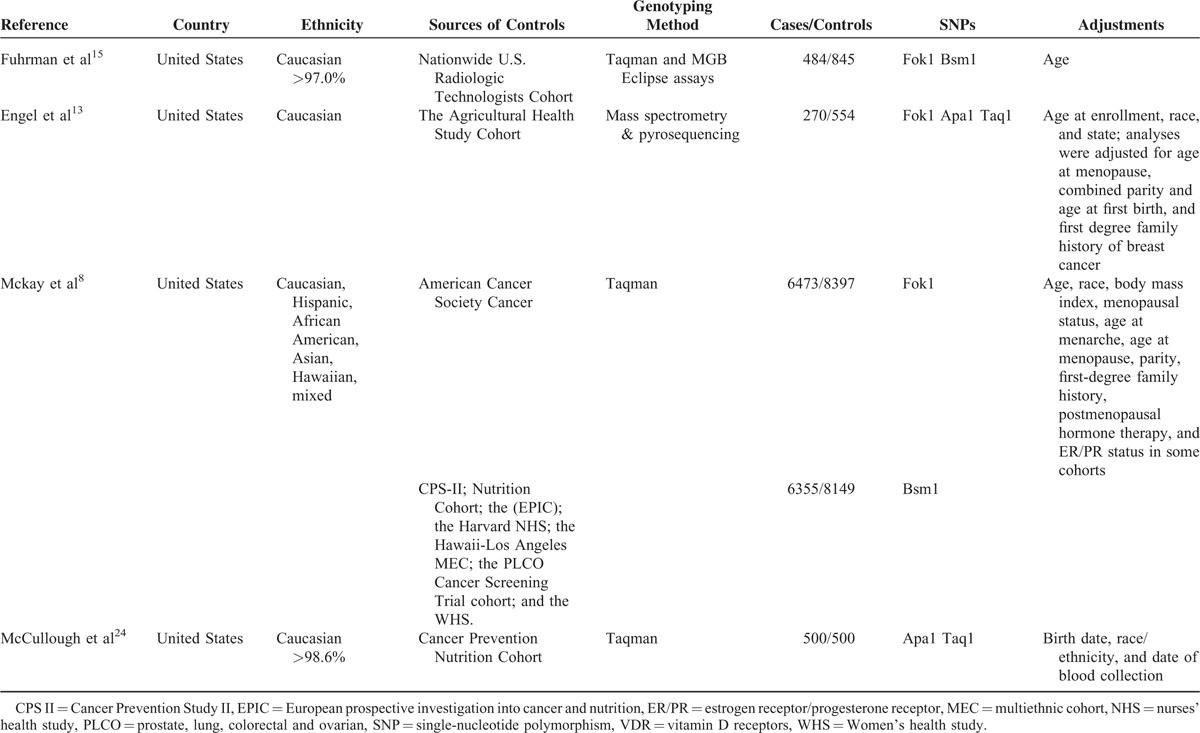
Study Features of VDR Polymorphism and Breast Cancer Risk in Meta-Analysis

### Quantitative Data Synthesis

The results of the associations between the Fok1, Apa1, Bsm1, and Taq1 polymorphisms and the breast cancer risk are shown in Table 2.

**TABLE 2:**
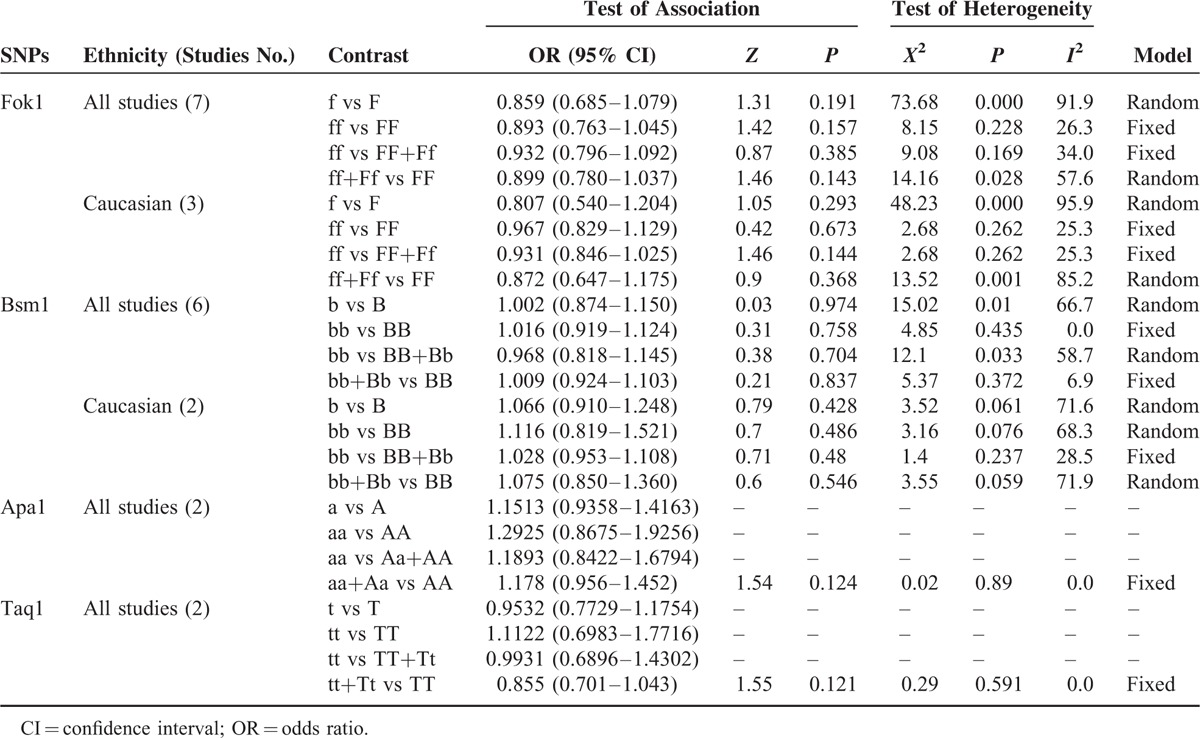
Summary ORs and 95%CI of the Associations Between the Fok1, Apa1, Bsm1, and Taq1 Polymorphisms and the Breast Cancer Risk

#### Fok1

**FIGURE 2:**
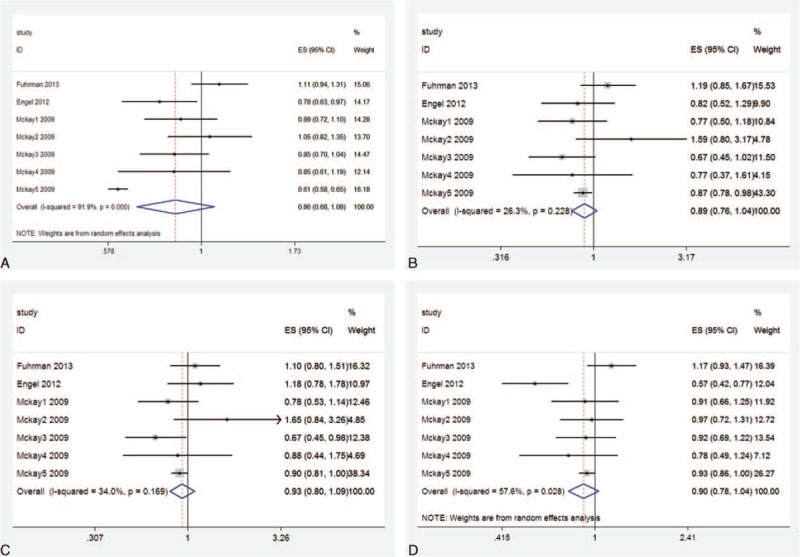
Forest plot and summary OR of the association between VDR Fok1 polymorphism and breast cancer risk. (A) Allele model (f vs F). (B) Homozygote model (ff vs FF). (C) Recessive models (ff vs FF+Ff). (D) Dominant models (ff+Ff vs FF). VDR = vitamin D receptor.

Meta-analysis of the 7 studies suggested that there was no association between allele contrast f versus F (OR: 0.859; 95%CI: 0.685–1.079), homozygote model ff versus FF (OR: 0.893; 95%CI: 0.763–1.045), recessive models ff versus FF+Ff (OR: 0.932; 95%CI: 0.796–1.092), and dominant models ff+Ff versus FF (OR: 0.899; 95%CI: 0.780–1.037) (Figure 2A–D). In the Caucasian ethnic subgroup, no association was found between allele contrast, recessive models, dominant models, and breast cancer risk.

#### Bsm1

There was no association between VDR Bsm1 polymorphism and risk of breast cancer, regardless of the allele contrast, recessive models, and dominant models. In the Caucasian subgroup, we also found no association.

#### Taq1 and Apa1

Only two studies (which consisted primarily [>95%] of Caucasians) studied the Taq1 and Apa1 polymorphisms. In the meta-analysis, the summary estimated for VDR polymorphism showed no significant association between Taq1 and Apa1 polymorphisms and breast cancer risk.

### Publication Bias

The Begg–Mazumdar test and Egger's test were performed to assess the publication bias. *P* >0.05 was observed among most of the genetic models of 4 polymorphisms except for Fok1, f versus F (shown in Table 3). Although Fok1, f versus F, showed publication bias under Egger's test but not on Begg–Mazumdar test and we strictly followed inclusion criteria and Venice interim criteria for protection of bias, therefore, we considered the results did not suggest any publication bias.

**TABLE 3:**
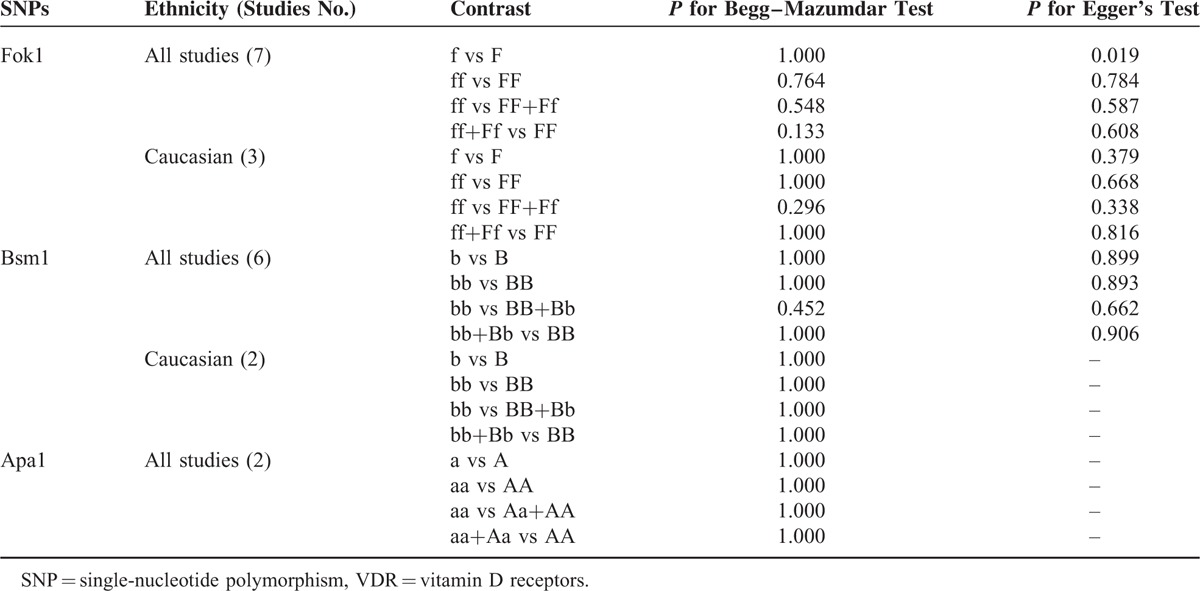
Egger's Test and Begg–Mazumdar Test for Four Polymorphisms of VDR

### Credibility of Meta-Analysis Results

The credibility of the association for each Fok1, Apa1, Bsm1, and Taq1 polymorphisms and the breast cancer risk are shown in Table 4. Most of the total frequency of the minor allele was >1000 except for Caucasian population of Fok1, ff versus FF, ff versus FF+Ff, general population of Apa1, aa+Aa versus AA, and Taq1, tt+Tt versus TT (sample sizes were between 100 and 1000). Therefore, category A or B was given for each result. Replication category varied from category A to C according to *I*^*2*^. The protection from bias category is A: there was a well-conducted inclusion criteria and protection of bias criteria. The overall scheme is shown in Table 4, which results in a characterization from “strong” evidence to “weak” evidence.

**TABLE 4:**
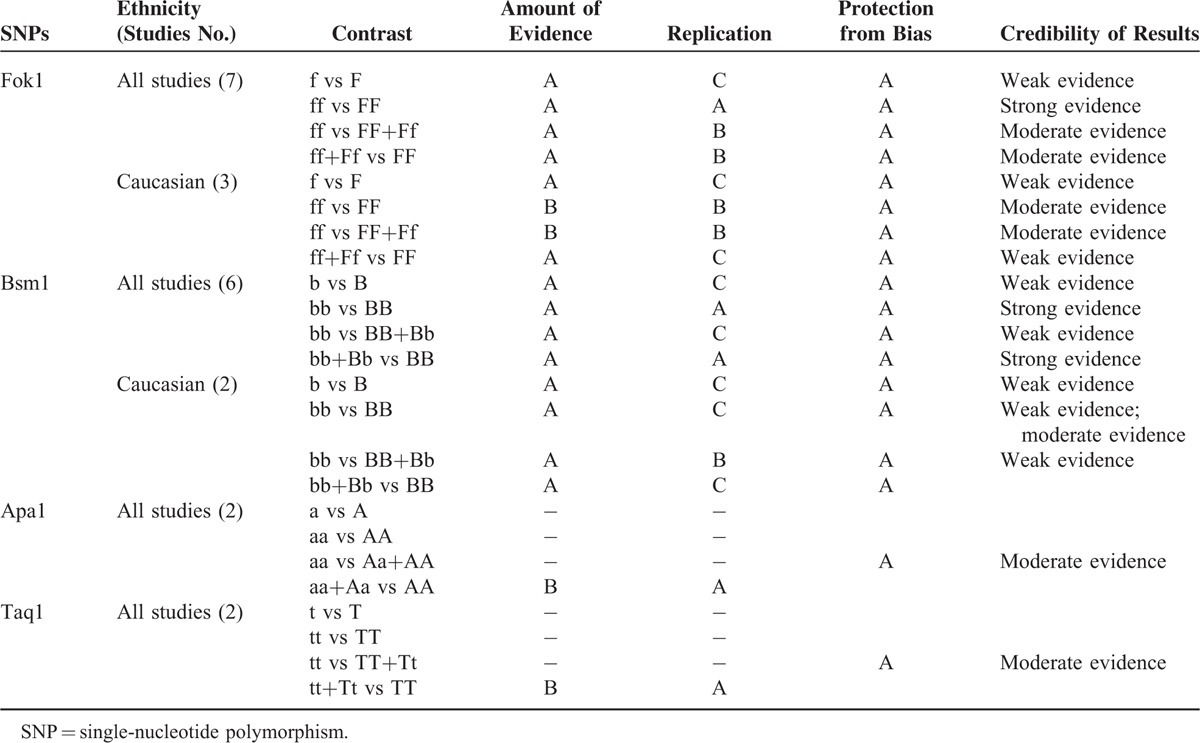
Credibility of the Association for Fok1, Apa1, Bsm1, and Taq1 Polymorphisms and the Breast Cancer Risk

## DISCUSSION

The pathogenesis of breast cancer remains unknown. It involves environmental factors, molecular signaling pathways, and host genetic factors. There is consistent epidemiologic evidence that increased vitamin D intake is associated with reduced risk of colorectal^25–28^ and breast cancers.^29,30^ The biologically active metabolite of vitamin D in vivo is 1,25-dihydroxyvitamin D which binds to VDR.^31^ The VDR gene is located on chromosome 12q12-q14, and several single-nucleotide polymorphisms (SNPs) have been identified that may influence cancer risk.^32^ Over the last two decades, a number of large population-based studies were carried out to investigate the association of variants in the VDR gene polymorphism and the risk of breast cancer. However, the results of these studies are controversial. The results of our meta-analysis from 8 prospective nested case-control studies indicated that there is no association between the SNPs in VDR (Fok1, Bsm1, Taq1, Apa1, and Poly A) and risk of breast cancer both in mixed races and Caucasian population.

In our meta-analysis, Fok1 polymorphism showed no association with breast cancer risk. The result is consistent with the previously meta-analysis, such as Huang et al and Xu et al.^33,34^ However, Zhang and Song, Wang et al, and Tang et al reported that ff genotype of Fok1 is a risk factor of breast cancer .^35–37^ The reasons are as follows: first, Huang et al is an updated and more carefully selected study than Wang et al and Tang et al.^33,36,37^ Second, the meta-analysis of Zhang and Song divided the article Mckay et al into 6 independent studies^8,35^ and our analysis of Mckay et al only included 5 different populations because one was the overlapping data.^8^ What's more, the Zhang and Song included both Mckay et al and Chen et al, which were from the same cohort and contained overlapping data.^8,^^35,38^ Third, our meta-analysis combined the prospective studies to overcome the shortcoming of retrospective studies on study population selection bias. Our study results in no association between Bsm1, Taq1, and Apa1 polymorphisms and breast cancer risk in mixed races. Previous meta-analysis's pooled ORs were similar to ours.^39^ Our study only pooled the prospective studies that are more reliable.

It is well established that VDR genotypes vary widely by ethnicity.^40^ In subgroup analyses, we conducted meta-analysis in Caucasian population. The results remained the same. There is no observed association between Fok1 and Bsm1 polymorphisms and breast cancer risk.

Our study had some strong points. First, all of the previous meta-analyses on the association collected both retrospective and prospective studies. In order to reduce the likelihood of selection bias our meta-analysis enrolled prospective studies only. Second, all controls’ SNPs genotype distributions were in HWE. Third, all utilized studies were strictly consistent with inclusion criteria. Fourth, no publication bias was observed indicating that the results might be unbiased.

As in any study, some limitations of this study should be considered. First, variant adjusted factors of ORs in each study such as age, age at menarche, menopausal status, body mass index, hormone replacement treatment usage, family history, race, smoking, etc. were different from the original studies. These gene–environment interactions could bring bias and heterogeneity in our study. Therefore, a more precise analysis should be conducted if individual data are available, which could permit the same adjusted factors. Second, significant heterogeneity was observed in overall comparisons and subgroup analyses. Selection bias, although no publication bias was observed, is a possible major source of heterogeneity. Different background and variant adjusted factors of controls might be the main reason. Third, some “weak” evidences were concluded from Venice interim criteria. All those “weak” evidence were because of category C on replication. Therefore, in our meta-analysis, when *I*^*2*^ was larger than 40%, we used the random-effects method^21^ for pooling in order to enhance the credibility of the results. Fourth, because of limited published data, our results need to be considered with caution.

In conclusion, our study provides the evidence that VDR polymorphism (Fok1, Bsm1, Taq1, and Apa1) was not associated with the risk of breast cancer in general population as well as the Caucasian population. Further studies are necessary to clarify these results.
